# Effects of Exposure to Bisphenol A during Pregnancy on the Pup Testis Function

**DOI:** 10.1155/2019/6785289

**Published:** 2019-05-21

**Authors:** Qingtao Yang, Xuxia Sui, Junjun Cao, Caixia Liu, Shukai Zheng, Mian Bao, Yuanni Huang, Kusheng Wu

**Affiliations:** ^1^Department of Preventive Medicine, Shantou University Medical College, Shantou 515041, Guangdong, China; ^2^Department of Urology, the Second Affiliated Hospital of Shantou University Medical College, Shantou, 515041, Guangdong, China; ^3^Department of Pathogenic Biology, Shantou University Medical College, Shantou, 515041, Guangdong, China

## Abstract

Testosterone plays an important prenatal role in male testis development. Bisphenol A (BPA) exposure during pregnancy affects testosterone levels and germ cell apoptosis of male pups, but little information is available for the mechanism. The aim of the present study was to investigate the mechanism by which BPA alters testosterone levels and germ cell apoptosis. Pregnant female C57BL/6J mice, throughout gestation, had access to drinking water containing BPA at 5 and 50 *μ*g/mL. Male pups were euthanized on postnatal days (PNDs) 1, 14, and 35. Relative to control, BPA exposure at 5 and 50 *μ*g/ml decreased testosterone level, as measured by chemiluminescent immunoassay, on PND14. Real-time PCR indicated mRNA levels for steroidogenic acute regulatory protein (StAR), cholesterol side-chain cleavage enzyme (CYP11A1), and 3-*β*-hydroxysteroid dehydrogenase/*△*-5-4 isomerase (3*β*-HSD) were significantly lower in the BPA pups compared to control. Additionally, BPA increased the percentage of TUNEL-positive seminiferous tubules, decreased the mRNA level of Bcl-2, and increased Bax expression, indicative of increased apoptosis. These results suggest that BPA exposure in utero decreases the testosterone concentration by decreasing steroidogenic enzymes (StAR, CYP11A1, and 3*β*-HSD). Furthermore, BPA exposure increases the apoptosis of germ cells, which is associated with proapoptotic changes in the levels of Bcl-2 and Bax.

## 1. Introduction

Bisphenol A (BPA) is widely used in a variety of consumer products, such as plastic bottles, food containers, and beverage cans, which can be ingested by humans because BPA is released from those products [1]. Similarly, composites and sealants used in dentistry can release BPA into saliva [2]. Additionally, BPA has been detected in maternal and fetal plasma, placental tissue, amniotic fluid, and umbilical cord blood in humans, indicating passage across the placenta during pregnancy [3–6]. Developing fetuses have a lower metabolic clearance and limited serum binding proteins, representing a higher risk of BPA exposure [3, 7, 8]. Therefore, we studied the male pups from maternal mice exposed to BPA during pregnancy.

Testicular Leydig cells are the predominant source of male testosterone. Data on the effect of BPA on testosterone is inconsistent. Exposure to high doses of BPA during pregnancy has been shown to decrease plasma testosterone at birth [9]. Perinatal exposure from gestational day (GD) 6 to postnatal day (PND) 20 to BPA at 4 mg/kg body weight (bw) increases plasma testosterone concentrations in adulthood [10]. Additionally, adult male rats perinatally exposed to BPA (1.2 and 2.4 *μ*g/kg bw) also possess increased serum testosterone levels [11]. In one instance, exposure of male rats to BPA at 2.5 *μ*g/kg/day (GD12 to PND21) did not affect serum testosterone, but it decreased intratesticular testosterone concentrations in adult animals [12]. Nevertheless, the mechanism of in utero exposure to BPA-induced alterations of serum testosterone is unclear. Therefore, one aim of the present study was to determine whether BPA exposure is associated with steroidogenic enzyme proteins. Testosterone withdrawal induces germ cell apoptosis [13, 14]. Moreover, early life exposure to BPA results in aberrant testicular function in adulthood, including a decrease in daily sperm production [15, 16], inhibition of testicular steroidogenesis [12], and disturbed spermatogenesis [17]. Therefore, the second aim of this study was to determine whether the BPA exposure during pregnancy affects germ cell apoptosis and the mechanism thereof.

Therefore, we hypothesize that exposure to BPA in C57BL/6J mice during pregnancy decreases testosterone concentration, and if so, to identify whether it is associated with the steroidogenic enzymes. Those enzymes mainly included steroidogenic acute regulatory protein (StAR), cholesterol side-chain cleavage enzyme (CYP11A1), and 3-*β*-hydroxysteroid dehydrogenase/*△*-5-4 isomerase (3*β*-HSD). Real-time PCR and immunohistochemistry were used to detected the levels of mRNA and localization of steroidogenic enzymes. TUNEL staining was used to detect apoptosis of germ cells, and the associated apoptotic mechanisms were also studied.

## 2. Materials and Methods

### 2.1. Reagents and Antibodies

BPA (CAS no. 80-05-7) was from Sigma Chemical Company (Saint Louis, MO, USA). Rabbit anti-CYP11A1 (ab175408), 3*β*-HSD (ab65156), StAR (ab203193), Bcl-2 (ab59348), and Bax (ab53154) primary antibody and secondary antibody anti-rabbit were from Abcam (Cambridge, MA). The DeadEndTM Colorimetric TUNEL System was from Promega Corporation (Promega, Madison, WI, USA).

### 2.2. Animals

Eight-week-old male C57BL/6J mice were purchased from Beijing Vital River Laboratory Animal Technology Co., Ltd. (Beijing, China). They were bred in an air-conditioned room with a 12-h light and 12-h dark cycle and had free access to water and a standard laboratory diet in the Laboratory Animal Center of Shantou University Medical College. Animals were allowed to acclimatize for 1 week, and females cohabited with males on a 1:1 basis. The morning on which a vaginal plug was used was regarded as GD1. Pregnant females were housed singly and received BPA at concentrations of 5 *μ*g/mL and 50 *μ*g/mL in drinking water (1% ethanol solution) from GD1 to the end of gestation. Control females received water containing 1% ethanol. One pregnant female consumed water at about 5 mL/day/mouse and the average body weight was about 25 g/mouse. Therefore, the mean level of BPA consumed by pregnant females was approximately 1 or 10 mg/kg/day, if all water from the bottle was considered to be consumed. For BPA, the lowest dose studied for risk assessment purposes was 50 mg/kg/day, which is the currently accepted lowest observed adverse effect level [18]. BPA concentrations used in this study are below the reference dose (50 mg/kg/day).

Spermatogenesis undergoes three successive phases: the mitosis of spermatogonia, the meiosis of spermatocytes, and the generation of spermatozoa [19]. The testes of mouse enrich gonocytes/spermatogonia on PND1. Meiosis of spermatocytes initiates on PND10, and pachytene spermatocytes are approximately one-third of the total cells in the seminiferous tubules on PND14 [20]. It takes about 35 days to complete the first round of spermatogenesis [21]. Therefore, PNDs 1, 14, and 35 were chosen in this study. Date of birth was designated as PND1. Pups were weaned on PND21. Pups born from control females were used as a control group. Body weights were measured on PNDs 1, 14, and 35. Animals were anesthetized with isoflurane. Pup blood was collected from the heart on PNDs 14 and 35, and the testes were removed for immunohistochemistry and real-time PCR analysis. At least three pups from different dams per time point were used. All experiments were approved by the Animal Research Ethics Committee of Shantou University Medical College.

### 2.3. Immunohistochemistry

Paraffin-embedded testes were sectioned into 4-*μ*m-thick pieces. Sections were dewaxed and hydrated and then blocked with 10% bovine serum albumin (BSA) in humidified chamber for 30 min. Sections were washed in phosphate-buffered saline (PBS) and incubated overnight at 4°C with primary antibodies (all primary antibodies were used at 1:200 dilution). Then, sections were washed and incubated with peroxidase-labeled secondary antibody for 1 hour at room temperature. Subsequently, sections were incubated in PBS containing 3,3-diaminobenzidine tetrahydrochloride (DAB, 0.5 mg/mL) for 2–5 min. Hematoxylin was used to counterstain and sections were photographed by a microscope Axio Imager A2 (Zeiss, Oberkochen, Germany). For negative controls, primary antibody was replaced with PBS and showed no positive reactions.

### 2.4. Real-Time PCR

Total RNA was isolated from the testes with TRIzol reagent (Invitrogen, Carlsbad, CA). Total RNA was reverse-transcribed into single-stranded cDNA using an RT reagent kit (TaKaRa, Shiga, Japan, cat. #RR047Q). Real-time PCR reactions were performed using an ABI Prism 7500 (Applied Biosystems) and TB Premix Ex Taq (TaKaRa, Shiga, Japan, Code No.RR820A). The amplification program was 30 s at 95°C, 5 s at 95°C, and 34 s at 60°C for 40 cycles. Real-time PCR primer sequences are listed in Table 1. Each sample was normalized on the basis of its GAPDH content.

### 2.5. Testosterone Measurement

Serum from hearts was centrifuged at 3000g for 5 min. Serum testosterone levels were measured by a chemiluminescent immunoassay (Beckman Coulter Inc., CA, USA) using a Beckman DXI 800 Analysis System (Fullerton, CA, USA).

### 2.6. TUNEL Staining

Apoptosis was performed by the terminal deoxynucleotidyl transferase (TdT)-mediated dUTP nick end labeling (TUNEL) method according to the manufacturer's protocol (Promega, Madison, WI, USA, cat. #G7130). Briefly, sections from paraffin-embedded testes were deparaffinized and rehydrated and incubated with 20 *μ*g/mL proteinase K solution for 15 min. Then sections were incubated with a mixture of TdT solution in a humidified chamber at 37°C for 60 min. The reaction was terminated by TdT Stop Buffer. Endogenous peroxidase activity was then blocked with 0.3% H_2_O_2_ for 5 min. Finally, sections were incubated with streptavidin-conjugated horseradish peroxidase diluted in PBS (1:500) for 30 min. Apoptotic cells showed brown-stained nuclei following incubation with DAB. Hematoxylin was used to counterstain. For a negative control, sections were incubated with the TdT reaction solution in the absence of TdT Enzyme and showed no positive reactions. All images were captured using a microscope Axio Imager A2 (Zeiss, Oberkochen, Germany). The number of TUNEL-positive cells within a seminiferous tubule cross-section was counted. The apoptosis-positive seminiferous tubule is considered as the seminiferous tubules containing more than three apoptotic germ cells. The apoptotic indices were calculated as the percentage of apoptosis-positive seminiferous tubules of the total tubules counted in a cross-section [24]. For each group, TUNEL-positive seminiferous tubules from three pups were counted on testis sections.

### 2.7. Statistics

A one-way ANOVA was used to determine statistical significance for pups from BPA treated females using the control pups from control females as a reference. Female pups were used for other experiments. Data are presented as the mean±standard deviation (SD). A* p*<0.05 was considered statistically significant. Graphs were plotted with* GraphPad Prism* 7.0 (San Diego, CA, USA). SPSS statistical software (Version 24.0; IBM SPSS Inc., Chicago, IL, USA) was used for all statistical analyses.

## 3. Results

### 3.1. Effect of BPA on Body Weights

Body weights of male and female pups were measured on PNDs 1, 14, and 35 (Table 2). Exposure to BPA at 50 *μ*g/mL (1.28±0.13 g) decreased the body weight of pups compared to controls (1.37±0.10 g) on PND1. Mean body weights of pups exposed to BPA at 5 and 50 *μ*g/mL were 6.97±0.73 and 6.84±0.50 g, respectively, which were lower than the control (7.43±0.69 g) on PND14. No significant difference was observed in mean body weight between BPA-exposed pups and control on PND35.

### 3.2. Effect of BPA on Serum Testosterone Concentration

To investigate whether BPA could affect the activity of mouse Leydig cells, the testosterone concentration in the plasms of male pups was measured (Table 3). There was a decrease in serum testosterone from 7.22±0.68 *μ*g/L in control to 5.21±0.21 and 4.43±0.24 *μ*g/L in the BPA-exposed pups at 5 and 50 *μ*g/mL, respectively, on PND14. Exposure with BPA at 50*μ*g/mL decreased testosterone concentration to 3.22±0.04 *μ*g/L compared with control (5.03±0.63 *μ*g/L) on PND35. These results indicate that steroidogenesis in the Leydig cells is reduced.

### 3.3. Effect of BPA on Steroidogenic Enzymes

**Figure 1:**
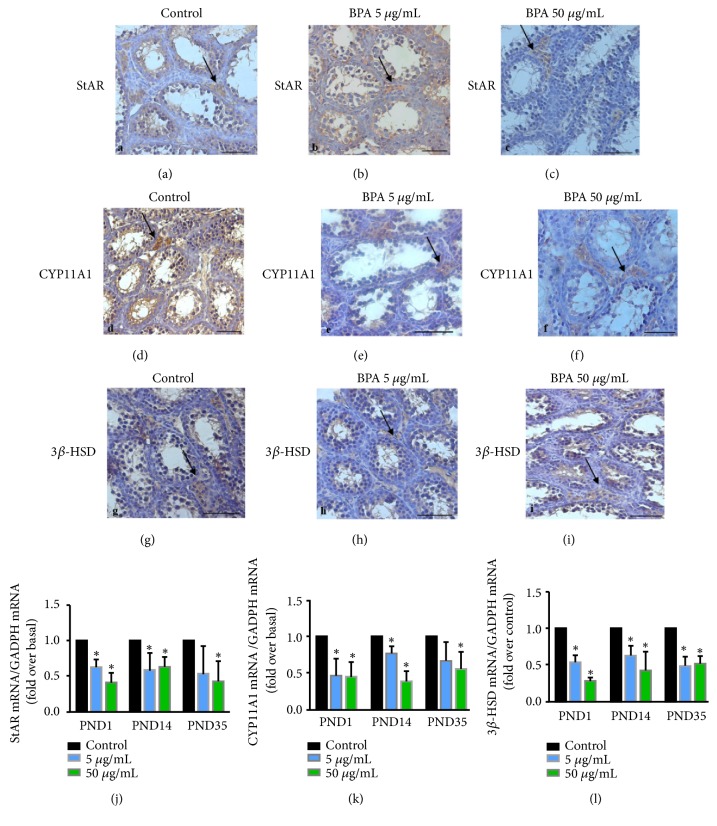
*Immunohistochemistry and real-time PCR analysis for steroidogenic enzymes in testes of pups from female exposure to BPA at 5 μg/mL or 50 μg/mL throughout gestation.* Immunohistochemical staining for StAR, CYP11A1 and 3*β*-HSD in control and BPA- exposed pups on PND14. (a–c) Immunolocalization of StAR in the testis. (d–f) Immunolocalization of CYP11A1 in the testis. (g–i) Immunolocalization of 3*β*-HSD in the testis. (j) Real-time PCR results of StAR in the testis. (k) Real-time PCR results of CYP11A1 in the testis. (l) Real-time PCR results of 3*β*-HSD in the testis. Data present mean ± SD. N=3 per treatment group, with each n representing a different litter from a different female. Black arrow points to Leydig cells that show positive staining. The ∗ indicates a significant difference compared to control at* P *< 0.05. Bar = 50 *μ*m.

We further detected the levels of the steroidogenic enzymes (StAR, CYP11A1 and 3*β*-HSD) in the testis using immunohistochemistry and real-time PCR (Figure 1). Immunohistochemistry was used to localize these proteins on PND14. Positive reactions for 3*β*-HSD, CYP11A1, and StAR were observed mainly in Leydig cells with or without BPA exposure (Figures 1(a)–1(i)).

Real-time PCR was used to detect mRNA levels (Figures 1(j)–1(l)). Relative to control, BPA exposure decreased the mRNA levels for StAR on PNDs 1 and 14, and only BPA at 50 *μ*g/mL decreased the mRNA levels for StAR on PND35. Similarly, BPA exposure decreased the mRNA levels for CYP11A1 on PNDs 1 and 14, and only BPA exposure at 50 *μ*g/mL decreased the mRNA levels for CYP11A1 on PND35 when compared with control. The expression of 3*β*-HSD decreased when exposed with BPA at 5 and 50 *μ*g/mL on PNDs 1, 14, and 35. Therefore, clear decreases in steroidogenic enzymes (StAR, CYP11A1, and 3*β*-HSD) were apparent on PNDs 1 and 14. Results indicate that testosterone production of Leydig cells in BPA-exposed mice was reduced.

### 3.4. Effect of BPA on Germ Cell Apoptosis

**Figure 2:**
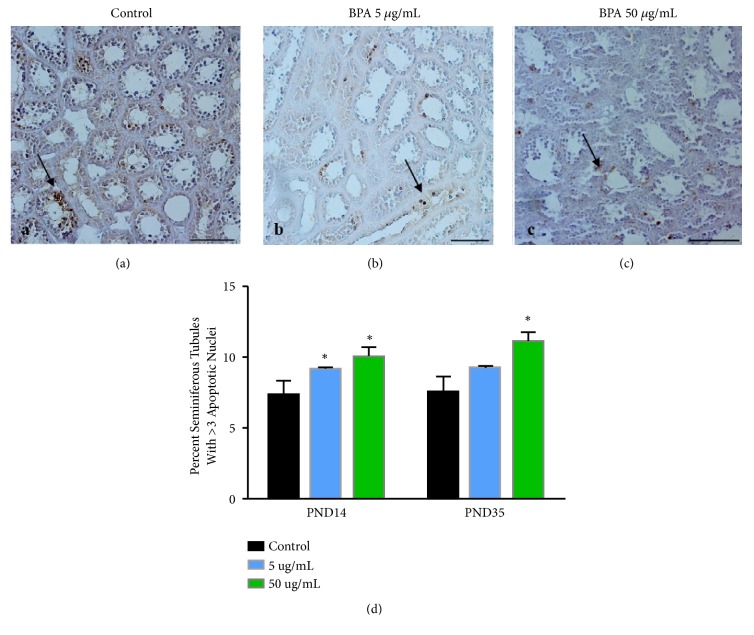
*Germ cell apoptosis in pups from female exposure to BPA at 5 μg/mL or 50 μg/mL throughout gestation*. Representative images of TUNEL staining in the testis for control (a), BPA at 5 *μ*g/mL (b) and BPA at 50 *μ*g/mL (c) on PND14. (d) The percentage of TUNEL-positive seminiferous tubules with greater than 3 apoptotic cells is represented as mean ± SD. N=3 per treatment group, with each n representing a different litter from a different female. The ∗ indicates a significant difference compared to control at* P* < 0.05. Bar = 100 *μ*m.

It was reported that testosterone withdrawal induced the germ cell apoptosis, and Bcl-2 family member is reported to be expressed in mammalian testes [13, 14]. Therefore, TUNEL staining was performed to detect the incidence of apoptosis in the testis of BPA-exposed male pups on PNDs 14 and 35 (Figure 2). The positive cells for TUNEL staining were mainly localized in seminiferous tubules of the testis (Figures 2(a)–2(c)). Compared with control, the percentage of TUNEL-positive seminiferous tubules dramatically increased after BPA exposure at 5 and 50 *μ*g/mL on PND14 (Figure 2(d)). On PND35, only BPA exposure at 50 *μ*g/mL increased the percentage of TUNEL-positive seminiferous tubules.

**Figure 3:**
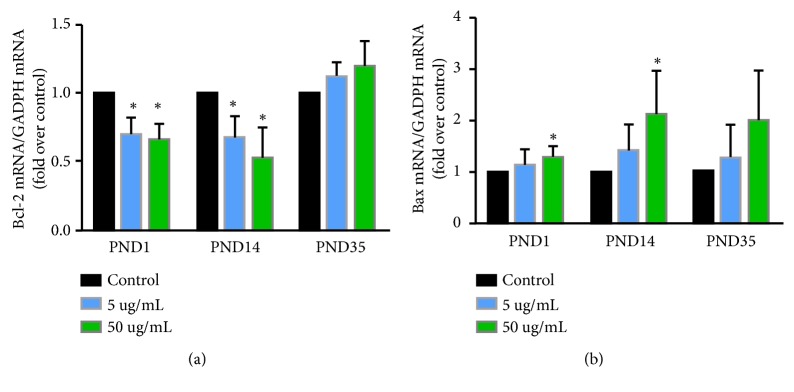
*Real-time PCR was conducted on the mRNA levels of Bcl-2 (a) and Bax (b)*. Data represent mean ± SD. N=3 per treatment group, with each n representing a different litter from a different female. The ∗ indicates a significant difference compared to control at* P* < 0.05.

To explore the mechanism of this apoptosis, real-time PCR was performed to detect Bcl-2 family member Bcl-2 and Bax (Figure 3). Bcl-2 proteins have an antiapoptotic effect, and Bax is known to promote apoptosis. BPA exposure decreased the mRNA levels for Bcl-2 on PNDs 1 and 14 (Figure 3(a)). However, only BPA exposure at 50 *μ*g/mL increased the mRNA levels of Bax on the same days (Figure 3(b)).

## 4. Discussion

Pups exposed to maternal BPA doses between 2.4 and 500 *μ*g/kg/day have shown increased postnatal body weight in different rodent species [25–28]. Prenatal exposure to BPA at 70 *μ*g/kg/day significantly increased the body weight of pups at birth, but differences were no longer apparent in adulthood [29]. The reasons for body weight gain may be the estrogenic action in nonreproductive tissues [12]. Perinatal exposure to BPA, from GD12 through weaning at PND21, did not affect body weights at 1, 21, 35, and 90 days of age [30]. However, some studies, using Sprague-Dawley rat and CD-1 mice, showed no significant alteration in pups exposed to a very wide range of maternal BPA doses between 0.001 and 5 mg/kg/day, while body weight of pups decreased if pups in utero were exposed to higher doses of BPA between 50 and 600 mg/kg/day [31, 32]. Our data show that exposure to BPA at 5 and 10 *μ*g/mL in drinking water (about 1 and 10 mg/kg/day) reduced the body weight of pups on PND14 but not on PND35 (Table 2). Therefore, it seems that BPA exposure does not cause sustainable changes in body weight.

Leydig cells are the main producers of androgens in male gonads. In cultured human Leydig cells, BPA decreases testosterone secretion [33]. However, in rat and mouse Leydig cells, only the highest BPA concentration (10^−5^ mol/L) decreased testosterone production, while concentrations equal to or lower than 10^−7^ mol/L had no effect [33]. Perinatal exposure (from GD12 through weaning at PND21) to BPA decreased intratesticular testosterone levels at PNDs 21, 35, and 90, but it did not decrease serum testosterone levels [30]. BPA did not alter serum hormone levels in rats exposed during gestation and lactation [34]. When mice are exposed to BPA at gestation days 10–16, serum testosterone levels do not change significantly in the adult male mouse [35]. Our study shows that BPA exposure significantly decreases serum testosterone on PND14, and only BPA exposure at 50 *μ*g/mL significantly decreased testosterone concentration on PND35 (Table 3). Therefore, it seems that the BPA effects on testosterone concentration are affected by the dose, time, duration of exposure, and detection age [12].

The mechanism by which BPA alters testosterone concentration is unknown. Testosterone is mainly produced in the Leydig cells of the testes. Under stimulation, cholesterol is mobilized by steroidogenic acute regulatory protein (StAR) to the mitochondrial inner membrane, where the cholesterol is converted to pregnenolone by the cytochrome P450 cholesterol side-chain cleavage enzyme (CYP11A1, also known as P450scc) [36–38]. Pregnenolone in smooth endoplasmic reticulum is next metabolized to androgens by a series of steroidogenic enzymes, including 3*β*-hydroxysteroid dehydrogenase (3*β*-HSD) and 17*β*-hydroxysteroid dehydrogenase 3 (17*β*-HSD3) [39]. BPA-induced inhibition of androgen secretion is likely because of decreased 17*β*-HSD3 protein [30]. Leydig cells from the testes of 90-d-old rats were incubated with BPA and luteinizing hormone. They show that BPA decreases androgen biosynthesis through the decreased 17*α*‐hydroxylase‐C17,20‐lyase (P45017*α*) enzyme. StAR and other biosynthetic enzymes showed no alteration [12]. Our results show that the mRNA levels of StAR, CYP11A1, and 3*β*-HSD are lower after the BPA exposure in utero on PNDs 1 and 14 (Figure 1), indicating that the ability of the testis to produce testosterone is weaker in mice prenatally exposed to BPA, which explains the decreased serum testosterone concentration after BPA exposure.

Testosterone in men is important for the maintenance of spermatogenesis. Testicular germ cell apoptosis occurs normally and continuously throughout life [40, 41]. Our results showed that exposure to BPA results in enhanced apoptosis in the seminiferous tubules instead of Leydig cells (Figure 2). This is supported by studies showing that exposure to BPA in the perinatal period increases the number of Leydig cells because of the increased proliferative activity in the testis of adult rats [30, 42]. In addition, testosterone acts as a survival factor for male germ cells and can reduce apoptosis in the testes of immature hypophysectomized rats [43]. The change in testosterone results in massive testicular germ cell loss [44]. However, it has also been reported that germ cell apoptosis does not change in the adult testis following in utero exposure to either 50 *μ*g/kg BPA or 1,000 *μ*g/kg BPA [35]. Our study shows that BPA exposure increases the percentage of TUNEL-positive seminiferous tubules (Figure 2), indicating that BPA exposure increases germ cell apoptosis. The increased apoptosis probably is likely because of the decreased testosterone concentration.

Apoptosis occurs in response to testosterone withdrawal, and Bcl-2 family member is reported to be expressed in mammalian testes [13, 14]. In the family of Bcl-2 proteins, some of them inhibit while others promote apoptosis. Bcl-2 proteins have an antiapoptotic effect. Overexpression of Bcl-2 in transgenic mice prevents germ cell apoptosis [45, 46]. Bax, another Bcl-2 family member, is known to promote apoptosis and is highly expressed from birth until the 4th week, but it becomes barely detectable in the adult testis [47]. Targeted gene disruption of Bax results in hyperplasia of spermatogonia in mice [48], and upregulation of Bax induces germ cell apoptosis in vitro [49]. Here, BPA exposure at 50 *μ*g/mL results in decreased mRNA levels of Bcl-2 and increased Bax on PND14 (Figure 3), which partially corresponds to the trend in apoptosis. This suggested that apoptosis induced by BPA exposure were associated with the levels of Bcl-2 and Bax.

## 5. Conclusions

Male and female pups exposed perinatally to BPA, through maternal ingestion of drinking water containing 5 and 50 *μ*g/mL BPA, display significantly lower body weight than the control pups on PND14, but no significant difference is observed on PND35. BPA exposure decreases serum testosterone on PND14, but only the 50*μ*g/mL BPA concentration was able to maintain decreased testosterone concentrations through PND35. We further demonstrate that the mRNA levels of the steroidogenic enzymes (StAR, CYP11A1, and 3*β*-HSD) are significantly lower in BPA-exposed mice compared to control on PNDs 1 and 14, indicating that the reduced testosterone concentrations following BPA exposure in utero may be a result of the decreased testosterone production capacity of Leydig cells. Moreover, BPA exposure also increases the percentage of TUNEL-positive seminiferous tubules on PND14 and is associated with decreased Bcl-2 and elevated Bax mRNA levels. These results indicate that BPA exposure in utero disrupts testis function up to PND14. Further study is needed to demonstrate the effect of BPA exposure in utero in adult testis function.

## Figures and Tables

**Table 1:** Primers are used for real-time PCR analysis.

Gene	Forward primer	Reverse primer

CYP11A1	GACCATCCTCCTTATCAATCT	CTCCAGCCTTCAGTTCAC
StAR	GTCCTTATGGCTGCTTATCT	TCCTGAAGTTGTCCTCTGA
3*β*-HSD	CTGCTGCTGCTACTACTG	AGAACTGTGGAAGGATGGT
Bax	AGACAGGGGCCTTTTTGCTA^a^	AATTCGCCGGAGACACTCG^a^
Bcl-2	CTTTGAGTTCGGTGGGGTCA^a^	AGTTCCACAAAGGCATCCCA^a^
GAPDH	AACTTTGGCATTGTGGAAGG^b^	ACACATTGGGGGTAGGAACA^b^

a and b primers are from references [22, 23], respectively.

**Table 2:** Body weights of pups (g).

PND	Control (N)	BPA (5 *μ*g/mL) (N)	BPA (50 *μ*g/mL) (N)

1	1.37±0.10 (19)	1.37±0.11 (25)	1.28±0.13∗(28)
14	7.43±0.69 (24)	6.97±0.73∗(32)	6.84±0.50∗ (33)
35	18.70±1.69 (24)	19.02±1.97 (23)	18.71±2.04 (27)

Values are mean ± SD. ∗ *P*<0.05 vs. control.

**Table 3:** Serum testosterone concentration in male pups (*μ*g/L).

PND	Control	BPA (5 *μ*g/mL)	BPA (50 *μ*g/mL)

14	7.22±0.68	5.21±0.21∗	4.43±0.24∗
35	5.03±0.63	3.79±0.35	3.22±0.04∗

Values are mean ± SD (n=3 per group). ∗ *P*<0.05 vs. control.

## Data Availability

The data used to support the findings of this study are available from the corresponding author upon request.

## Abbreviations

StAR: Steroidogenic acute regulatory protein
CYP11A1: Cholesterol side-chain cleavage enzyme
3*β*-HSD: 3-*β*-Hydroxysteroid dehydrogenase/△-5-4 isomerase
BPA: Bisphenol A
PND: Postnatal days
GD: Gestational day
TUNEL: Terminal deoxynucleotidyl transferase (TdT)-mediated dUTP nick end labeling
PBS: Phosphate-buffered saline
DAB: 3,3-Diaminobenzidine tetrahydrochloride
17*β*-HSD3: 17*β*-Hydroxysteroid dehydrogenase 3
P45017*α*: 17*α*‐Hydroxylase‐C17,20‐lyase.
